# Is adenosine a modulator of peripheral vasoconstrictor responses?

**DOI:** 10.1007/s10286-016-0345-y

**Published:** 2016-02-05

**Authors:** Lior Dayan, Silviu Brill, Uri Hochberg, Giris Jacob

**Affiliations:** Pain Institute, Tel Aviv Medical Center and Sackler Faculty of Medicine, TAU, Tel Aviv, Israel; Recanati Autonomic Dysfunction Center Tel Aviv Medical Center and Sackler Faculty of Medicine, TAU, Tel Aviv, Israel; Internal Medicine Department, Tel Aviv Medical Center and Sackler Faculty of Medicine, TAU, Tel Aviv, Israel; Department of Medicine and Physiology, Faculty of Medicine, Weizmann 6, 64239 Tel Aviv, Israel

**Keywords:** Veno-arteiolar reflex, Adenosine, Myogenic, Vascular

## Abstract

**Background:**

Local vasoconstrictor reflexes, the vascular myogenic response (VMR) and the veno-arterial reflex (VAR) are necessary for the maintenance of regional blood flow and systemic arterial blood pressure during orthostatic stress. Their molecular mechanism is unknown. We postulated that adenosine is involved in the activation of these local reflexes.

**Methods:**

This hypothesis was tested in 10 healthy male volunteers (age 29 ± 3 years, BMI 24 ± 1 kg/m^2^). We used veno-occlusive plethysmography method for the assessment of forearm arterial blood flow at baseline and upon causing local venous congestion by inflating a second cuff to 40 mmHg for 4 min (VAR) and during placement of the forearm 40 cm below cardiac level for 4 min (VMR). These measurements were repeated after local infusion of either saline or aminophylline, non-selective adenosine blockers, using the Bier block method.

**Results:**

Rest baseline forearm blood flow was comparable in both arms. Saline did not affect the baseline forearm blood flow. However, aminophylline causes a significant increase in baseline forearm blood flow of 34 ± 6 % (*p* = 0.002). VAR demonstrated a decrease in forearm blood flow of 49 ± 4.5 % and after saline infusion it remained unchanged, 49 ± 5 % (*p* = 0.92). However, aminophylline causes significant decrease in the VAR by 35 ± 3 % (*p* = 0.02). But, both, saline and aminophylline did not affect the VMR.

**Conclusion:**

Arterial vasoconstriction triggered by venous congestion, which is the veno-arterial reflexis seems to be modulated by adenosine, at least partially. This “sensory” reflex requires further pharmacologic physiologic investigation.

## Introduction

Blood flow in an organ is directly proportional to its metabolic demand (requirements), and the regulation of organ blood flow is achieved by the concomitant release of neurally and locally derived vasoactive molecules [6]. Local vasoregulation is necessary for maintaining regional blood flow and systemic arterial blood pressure in physiological challenges. For example, it is estimated that about 40 % of the increase in systemic vascular resistance during orthostatic stress is due to local vascular responses which complement sympathetically driven responses [15].

The veno-arteriolar reflex (VAR) and the veno-arteriolar-myogenic response (VMR) participate and complement baroreflex-mediated sympathetic vasoconstriction in local vasoregulation [19]. The VAR is a local neural reflex that causes arterial vasoconstriction and is activated following venous congestion. The VAR is a genuine reflex because it requires an intact neuronal network and can be abolished by blocking sympathetic nervous transmission [14]. The VMR is an inherent pressure-sensitive myogenic response that is elicited in arterioles when transmural pressure changes and is generated independent of neural, metabolic, and hormonal influences [17]. When transmural pressure increases, vasoconstriction results and vasodilation ensues when transmural pressure is reduced. There is no experimental method for producing a pure myogenic response in the human vasculature. However, the VAR and the VMR can be easily demonstrated in the extremities of humans and animals. However, there is no general agreement on the importance of these reflexes, because of the limited standardized methodology for their investigation.

Adenosine, the end product of ATP hydrolysis and co-released with norepinephrine from sympathetic nerve terminals, is one of many locally derived vasoactive molecules that can regulate blood flow in an organ [5]. Adenosine can also inhibit ATP and norepinephrine release by stimulating presynaptic auto- and heteroreceptors [4, 16]. Adenosine exerts its action by stimulating four specific G-protein-coupled receptors: A_1_, A_2a_, A_2b_, and A_3_. Adenosine causes endothelial-dependent vasorelaxation by stimulating A_1_ and A_2_ receptors on the vascular endothelium and activating cyclic GMP and cyclic AMP signalling pathways in vascular smooth muscle [7]. In addition to this local vasoactivity, adenosine also has a number of actions that are considered to be neuromodulatory. Since the VAR is a neural reflex and adenosine is a local ubiquitous neuromodulatory molecule, we postulated that adenosine modulates the VAR and influences the VMR. In this report, we inform on the results of an investigation in which we tested this hypothesis in the forearm vasculature of ten human volunteers using aminophylline (AMINP), the non-selective adenosine receptor antagonist [3], and venous occlusion plethysmography.

## Materials and methods

### Subjects

The study was approved by the local ethics committee, and each subject signed a consent form to participate in the study following an explanation of its purpose, nature, and potential risks.

The study comprised ten healthy male subjects (age 29 ± 3 years [mean ± standard error of the mean], height 171 ± 2.6 cm; weight 71 ± 1.3 kg, and body mass index (BMI) 24 ± 1 kg/m^2^) with no known medical condition(s) and were not taking any prescription and/or non-prescription medications. The ten study participants were recruited using local community advertisements. Exclusion criteria were (1) history of any major trauma to the upper extremities (2) clinical evidence of peripheral vascular disease, and (3) history of allergy to any drug. Subjects were requested to avoid methylxanthine-containing beverages, such as coffee, tea, or cola drinks, and foods for at least 24 h before making any measurements because methylxanthines are competitive antagonists of adenosine receptors.

### Forearm blood flow measurements

Venous occlusion plethysmography was used to measure forearm blood flow. In this method, venous return from the forearm is intermittently interrupted by placing an inflatable cuff around the subject’s upper arm and then inflating the cuff to a pressure of 70 mmHg, which is higher than the venous blood pressure and lower than the diastolic blood pressure. The rate of increase in the forearm volume is an index of arterial inflow, and was determined using a mercury-in-silastic strain gauge (ECR5, DE Hokanson Inc., Bellevue, WA, USA). All measurements were made in each subject in a quiet, darkened, and air-conditioned room with an ambient temperature of ~24 °C.

#### Assessment of the VAR and the VMR

The VAR and the VMR were assessed using previously described protocols [17, 19]. To assess the VAR, a second, superficial cuff was placed over the deep inflatable cuff that is in contact with the skin and is used for blood flow measurement. The deep cuff was then inflated to a steady pressure of 40 mmHg for four minutes to produce continuous venous congestion, which in turn causes regional arteriolar vasoconstriction and reduced blood flow by activating the VAR. Blood flow is then measured by intermittently inflating the superficial cuff to 70 mmHg, a pressure which has been previously reported to be sufficient to provide additional transient venous occlusion and measure blood low [13, 19].

#### Measurement of the VMR

The VMR was generated in the forearm vasculature of each subject by placing the subject in a supine position to avoid systemic baroreflex changes. Blood flow was then measured during dependency of the forearm, which was 40 cm below the cardiac level for four minutes using a previously described protocol [15, 19]. This dependency increases static venous and arterial pressure by 31 mmHg, and concomitantly stimulates myogenic mechanisms in vascular smooth muscle to generate a veno-arteriolar response, namely local vasoconstriction and reduced blood flow. We must admit that this method, arm dependent, elicits also the VAR because of the venous congestion. A method that can assess the VMR alone also exists.

#### AMINP infusion

**Fig. 1:**
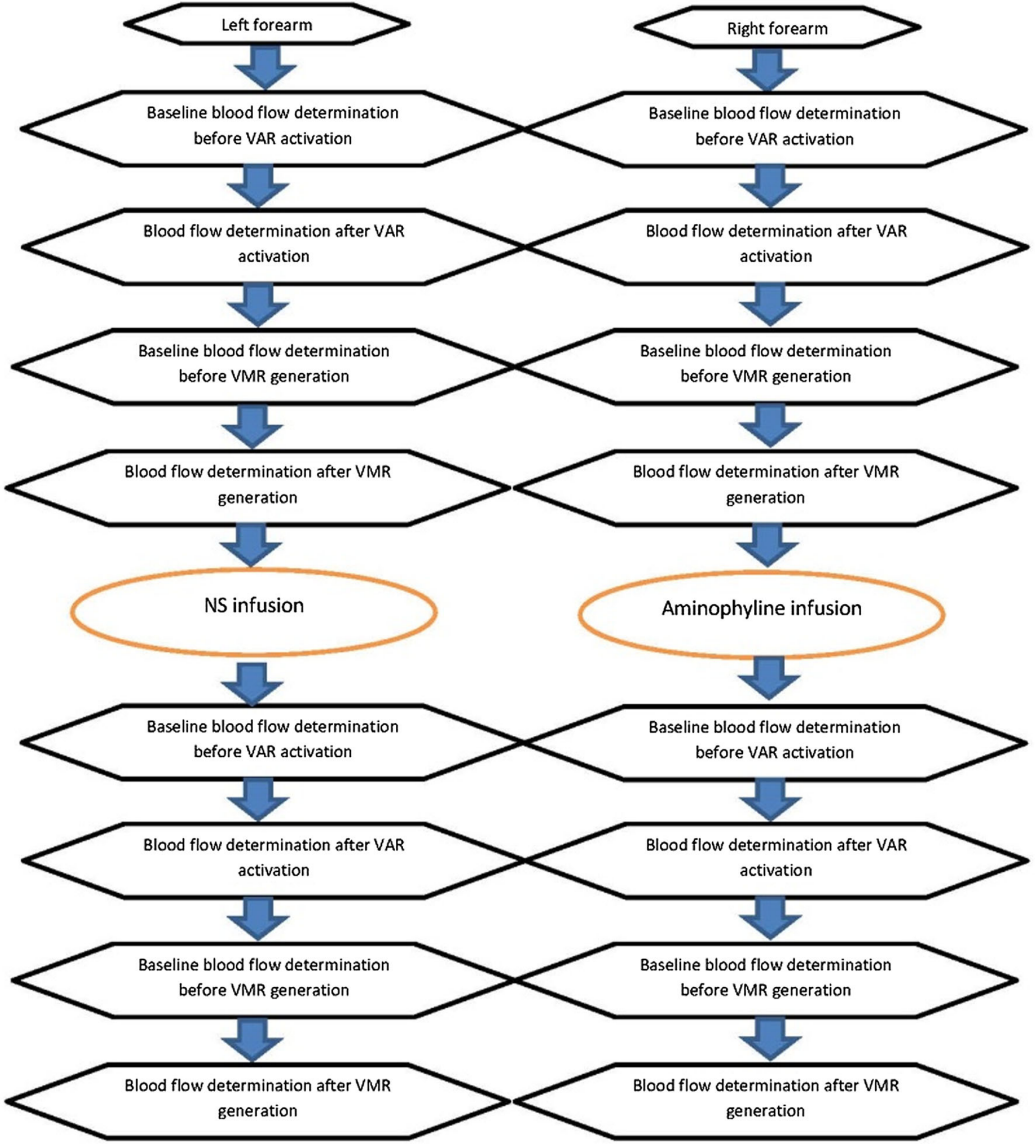
Flowchart of the experimental protocol for measuring blood flow, activating the veno-arteriolar reflex (VAR), and generating the veno-arteriolar-myogenic response (VMR) following an intravenous infusion of aminophylline (*right arm*) and a 0.9 % sodium chloride solution [normal saline (*NS*)] *(left arm*)

AMINP (right arm) or a 0.9 % sodium chloride solution (normal saline; NS) (left arm) was infused in a manner that is similar to that which is used for achieving regional anaesthesia of the upper forearm (modified Bier block), as described by Cui et al. [9] (Fig. 1).

#### Venous emptying of the left arm and local venous infusion of NS

After determining baseline blood flow and changes in blood flow after activating the VAR and generating the VMR in the left and right upper arms, the left arm was raised to a level above that of heart for 4 min to allow venous emptying. Before returning the arm to its supine position, the deep arm cuff was inflated to a pressure of 250 mmHg to avoid venous re-filling. NS (40 ml) was then infused through a dorsal wrist vein at a constant flow rate over four minutes. After a 10-min equilibration period, the deep cuff was then deflated to restore normal blood flow in the arm. Before making a second series of measurements in the same arm, a 6-min rest period was made to prevent the post-ischaemic hyperaemia that follows prolonged ischaemia and to ensure that the rate of blood flow returns to its baseline value after ischaemia.

#### Venous emptying of the right arm and local venous infusion of AMINP

After completing the assessment of the vascular responses in the left upper arm, a 30-min rest period was allowed before starting venous emptying of the right arm in the same manner that was described for the left arm. After venous emptying of the right arm, AMINP (40 mg dissolved in 40 ml NS) was then infused through a dorsal wrist vein at a constant flow rate over 4 min. This dose of AMINP was chosen because it has been previously reported that this dose can efficiently block adenosine receptors [9]. After measuring blood flow, activating the VAR, and generating the VMR, a second series of determinations was made after deflating the deep cuff, the 10-min equilibrium period, and the 6-min resting period, as previously described in the previous subsection.

#### Calculations and statistical analysis

All statistical analyses were done using Excel (Microsoft, Redmond, WA, USA) and a computerized statistical software package (GraphPad Prism, version 5; GraphPad Software, San Diego, CA, USA). The Wilcoxon matched-paired test was used to compare blood flow and the VMR in the left and right arms before and after activating the VAR. Blood flow was expressed as volume change in ml/100 mg tissue/minute. The changes in blood flow and the VMR following activating the VAR were normalized to the baseline blood flow before activating the VAR using the following equation: (1-X/BL) × 100, where X is the blood flow after the activating the VAR and BL is blood flow before activating the VAR. Data are presented as mean ± standard error of the mean (SEM), and statistical significance was set at 5 %.

## Results

The mean heart rate and blood pressures of the ten study subjects at baseline and following each treatment are displayed in the Table 1. These cardiovascular variables were not affected by the intravascular infusion of either NS or AMINP.

**Table 1:** Blood pressure (BP mm Hg), and heart rate (HR, bpm) and forearm blood flow (FBF ml/min/dl tissue), at baseline, after saline (NS) and aminophylline (AMINP) local infusion (modified Bier block) and during the assessment of VAR (veno-arterial reflex) and VMR (vascular myogenic response)

	Baseline	NS	AMINP
Systolic BP mm Hg	117 ± 1.5	118 ± 1.5	117 ± 1.5
Diastolic BP mm Hg	76 ± 1.5	75 ± 2	72 ± 2
HR bpm	67 ± 1	67 ± 3	68 ± 3
FBF ml/min/dl	2.5 ± 0.2	2.5 ± 0.2	3.1 ± 0.2
VAR-FBF	1.21 ± 0.09	1.23 ± 0.09	2.05 ± 0.12
FBF-VMR	1.57 ± 0.13	1.59 ± 0.2	1.85 ± 0.1

Values are displayed as Mean ± SEM

Baseline forearm blood flow in the left and right forearms were comparable (2.5 ± 0.2 ml/dL tissue (left forearm) and 2.3 ± 0.2 ml/dL tissue (right forearm), and this result indicates that extremity dominance does not affect blood flow.

**Fig. 2:**
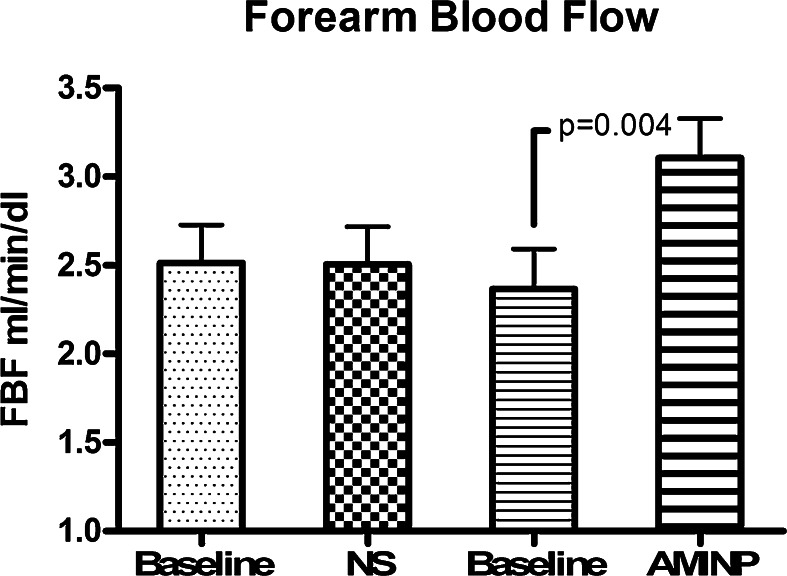
Changes in forearm blood flow before activating the veno-arteriolar reflex and following an intravascular infusion of normal saline (*NS*) or aminophylline (*AMP*) in the ten healthy male subjects. Values are displayed as mean ± standard error of the mean

Blood flow in the left arm did not change following the NS infusion (2.5 ± 0.2 ml/dL tissue before and after NS infusion, *p* = 0.98). However, blood flow in the right arm significantly increased (*p* = 0.03) before activating the VAR and following the AMINP infusion (2.36 ± 0.22 ml/dL tissue (before) versus 3.11 ± 0.22 ml/dL tissue (after); *p* = 0.03) (Fig. 2).

**Fig. 3:**
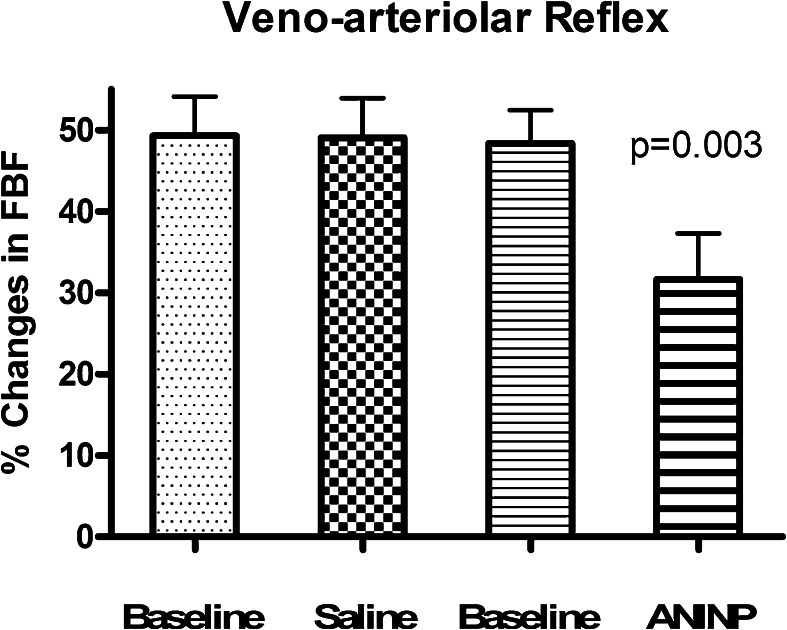
Changes in forearm blood flow after activating the veno-arteriolar reflex and following an intravascular infusion of normal saline (*NS*) or aminophylline (*AMP*) in the ten healthy male subjects. Values are displayed as mean ± standard error of the mean

Figure 3 displays the changes in forearm blood flow after activating the VAR and following the intravascular infusion of either NS or AMINP. In the left forearm, activating the VAR without and with the intravascular infusion of NS decreased forearm blood flow by 49.3 ± 4.5 % without the intravascular infusion of NS and by 49.1 ± 4.8 % with the intravascular infusion of NS. In the right forearm, activating the VAR without the intravascular AMINP infusion decreased blood flow by 48.4 ± 4 %. However, activating the VAR with the intravascular AMINP infusion decreased forearm blood flow by 35 ± 3 %. This reduction in blood flow was significantly less (*p* = 0.0027) than that caused by activating the VAR without the intravascular AMINP infusion.

**Fig. 4:**
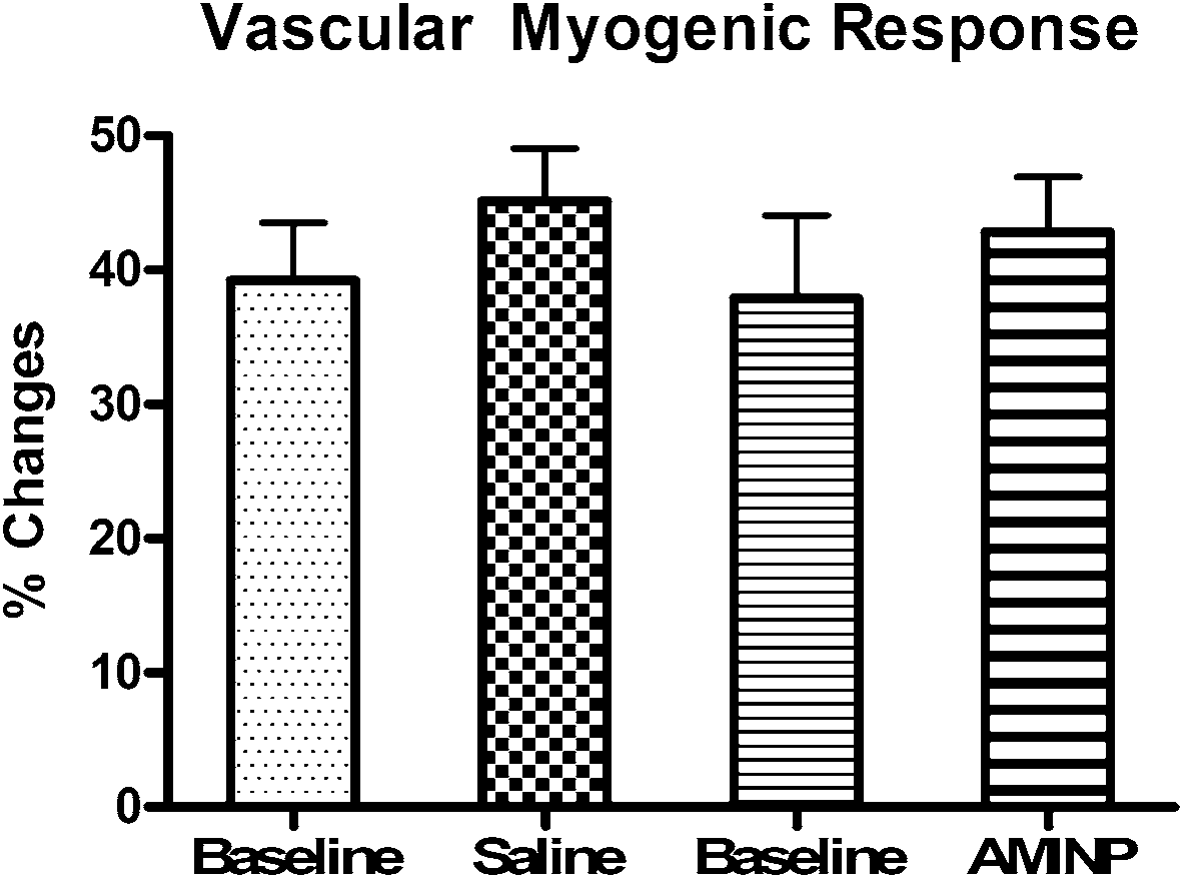
Changes in the FBF response to VMR generation before and after an intravascular infusion of normal saline (*NS*) or aminophylline (*AMP*) in the ten healthy male subjects. Values are displayed as mean ± standard error of the mean

Figure 4 displays the changes in VMR as measured by changes in forearm blood flow (FBF) before and after an intravascular infusion of normal saline (NS) or aminophylline (AMINP). Generation of the VMR in the left extremity without the intravascular NS infusion caused forearm blood flow to decrease to 39.2 ± 4.3 % of its baseline value. Following the intravascular NS infusion, generation of the VMR caused forearm blood flow to decrease by 45.1 ± 3.9 %, and this decrease was not statistically significantly different to the decrease in forearm blood flow without the NS infusion. Generation of the VMR in the right extremity caused forearm blood flow to decrease to 37.9 ± 6 % of its baseline value. Following the intravascular AMINP infusion, generation of the VMR caused forearm blood flow to decrease to 42.9 ± 4.1 % of its baseline value, and this decrease was not statistically significantly different to the decrease in forearm blood flow without the AMINP infusion.

## Discussion

In this study, we found that blocking adenosine receptors in the human forearm vasculature using AMINP diminishes the local vasoconstrictor response to a sympathetic-mediated reflex, namely the VAR, without affecting the VMR. This finding indicates that adenosine, in addition to its direct vasodilator action [7] is involved in the neuromodulation of the VAR.

In our experiment, NS and AMINP were infused intravenously in a manner that is similar to that used for achieving regional anaesthesia of the forearm. Due to the very low dose of AMINP and the method of local administration, systemic cardiovascular variables are not affected by AMINP. The absence of change in the systemic cardiovascular variables confirms that AMINP acted locally at the level of vascular wall and interstitium. We found that forearm blood flow increases following a local AMINP infusion before activating the VAR and generating the VMR. We attribute this increase in forearm blood flow to the mechanism of cyclic AMP action on blood vessels. AMINP inhibits phosphodiesterase activity and thereby increases the intracellular levels of cyclic AMP, which in turn causes smooth muscle cell relaxation and vasodilatation [18].

In the extremities of healthy humans, local vascular responses are generated in an orthostatic challenge and before activating other essential neuro-humoral processes with a similar effect, namely the autonomic nervous system (within 4–11 s) and renin–angiotensin–aldosterone axis, whose peak response occurs after 30 min. Local vascular responses can be divided into those which are completely dependent and those which are partially dependent on the sympathetic nervous system. The VAR is a genuine neuronal reflex whose activation is completely dependent on the sympathetic nervous system, whereas the VMR is a response which is mostly dependent on local vascular myogenic responses [6, 17, 19]. Evidence for the existence of this concept (i.e. that the VAR is neurogenic and VMR is myogenic in origin) comes from the results of studies which showed that a local anaesthetic infusion blocks the VAR but not the VMR. The results of studies by Henriksen et al. and Crandel et al. [8, 15] have revealed that different techniques are needed to activate the VAR and generate the VMR. Okazaki et al. extrapolated that the VAR also depends on the local perfusion pressure. General agreement exists on the VAR that it is a pure axonal reflex and can be activated by increasing venous congestion and pressure, while sparing the arterial system, and seems to be a local efferent pathway of the sympathetic nervous system. The VMR is mostly an intrinsic myogenic response which is mainly dependent on cellular mechanisms, such as the epithelial Na^+^ channel, EnaC, mechanosensitive ion channel [10].

By comparing the changes in forearm blood flow following stimulation of VAR or generation of the VMR, we were able to distinguish between a response that is completely neurogenic and a response that is mainly myogenic. In this study, we found that an intravascular AMINP infusion blunted the vasoconstrictor response following activation of the VAR. We also found that this AMINP infusion does not affect the VMR. From our results, we can deduce that adenosine blockade affects the neurogenic response rather than the myogenic vasoconstrictor response. Since the VAR is a sympathetically mediated reflex, we can also deduce that adenosine blockade modulates a sympathetic-dependent reflex. Specifically, adenosine blockade decreases the anticipated vasoconstrictor response following activation of the VAR.

It is also possible that vasoconstrictor reflex is mediated by adenosine because of its ability to stimulate the sympathetic system (the afferent signalling pathway) because it has been reported that adenosine can facilitate sympathetic activity [2].

Adenosine’s action on the vasculature is complex: it has direct and indirect vasodilator activity, as well as indirect vasoconstrictor activity. Its direct vasodilator activity is mediated by adenosine receptors which are located on vascular smooth muscle. The mechanism of adenosine’s indirect vasodilation is mediated by adenosine receptors on sympathetic nerve terminals, which are heteroreceptors and inhibit norepinephrine release [7]. If the vascular response following activation of the VAR was dependent upon stimulation of these heteroreceptors, we would have expected that antagonizing the adenosine receptors by AMINP would result in augmentation of the vasoconstrictor response following activation of the VAR. Since the vasoconstrictor response was attenuated following activation of the VAR and the AMINP infusion, we concluded that adenosine-mediated indirect vasodilation is not involved in the VAR. Adenosine is also an indirect vasoconstrictor and venous congestion activates an adenosine-dependent pathway that results in vasoconstriction. Engelstein et al. [12] reported that endogenous adenosine can increase sympathetic activity via its action on an afferent neuronal pathway. Since we showed that adenosine blockade attenuated the sympathetic-mediated vasoconstriction following activation of the VAR, we concluded that adenosine is an important modulator of the afferent limb of the VAR.

Our finding of adenosine receptor-mediated facilitation of a local sympathetic reflex may have clinical significance. We recently reported that the vasoconstrictor response following activation of the VAR, but not following generation of the VMR, is exaggerated in the affected extremity of patients with chronic regional pain syndrome 1 (CRPS1), a common painful disorder of an extremity which may develop as a disproportionate consequence of a minor trauma [11]. In addition to pain, autonomic (sympathetic) disturbances are characteristic clinical signs of this disorder [1]. Since we found that the VAR is modulated and facilitated by adenosine, we suggest that adenosine antagonists could be used in the treatment of patients with CRPS1.

## Study limitations

Cyclic adenosine monophosphate (cAMP) is broken down by a cAMP-dependent phosphodiesterase and inhibiting this phosphodiesterase can cause vasorelaxation because cAMP inhibits myosin light chain kinase, the enzyme responsible for phosphorylating smooth muscle myosin and vasocontraction. If the effect of aminophylline on the VAR is due to this inhibitory action on cAMP-dependent phosphodiesterase, we would have expected the VMR to be attenuated in this investigation. Since we did not find such attenuation, we are confident that blockade of adenosine receptors is the underlying mechanism of aminophylline’s action in this investigation.

When measuring the magnitude of the vascular response following activation of the VAR, we normalized the blood flow values following the aminophylline infusion to the baseline blood flow values. This normalization was performed because of the inhibitory action of aminophylline on cAMP-dependent phosphodiesterase that results in vasodilation.

Another limitation of our study is that we did not measure the blood levels of aminophylline after its infusion. Since we did not observe any systemic effects following the very low dose of local intravenous AMINP infusion, we concluded that the effects of aminophylline are local rather than systemic.

The generation of VMR includes activation of the VAR, at least partially, because of the used limited method, as we previously stated.

## Conclusions

Local blockade of adenosine receptors in the forearm vasculature inhibits the local vasoconstrictor reflex, VAR, but not the VMR. Our results indicate that adenosine potentiates the local vascular responses by activating the VAR without influencing the generation of the VMR following venous congestion. Future investigation is required to identify the specific subtypes of adenosine receptors that are involved in these local vascular responses.

## Abbreviations

AMINP: Aminophylline
CRPS1: Chronic regional pain syndrome 1
NS: Normal saline
VAR: Veno-arteriolar reflex
VMR: Veno-arteriolar-myogenic response
